# GeneSetCluster: a tool for summarizing and integrating gene-set analysis results

**DOI:** 10.1186/s12859-020-03784-z

**Published:** 2020-10-07

**Authors:** Ewoud Ewing, Nuria Planell-Picola, Maja Jagodic, David Gomez-Cabrero

**Affiliations:** 1 Department of Clinical Neuroscience, Center for Molecular Medicine, Karolinska Institutet, 171 77 Stockholm, Sweden; 2 Translational Bioinformatics Unit, Navarrabiomed, Complejo Hospitalario de Navarra (CHN), Universidad Pública de Navarra (UPNA), IdiSNA, Pamplona, Spain; 3 Unit of Computational Medicine, Department of Medicine, Solna, Center for Molecular Medicine, Karolinska Institutet, 171 77 Stockholm, Sweden

**Keywords:** Data-mining, Gene-set enrichment, Clustering pathways, Overlapping pathways, Clustering gene-sets

## Abstract

**Background:**

Gene-set analysis tools, which make use of curated sets of molecules grouped based on their shared functions, aim to identify which gene-sets are over-represented in the set of features that have been associated with a given trait of interest. Such tools are frequently used in gene-centric approaches derived from RNA-sequencing or microarrays such as Ingenuity or GSEA, but they have also been adapted for interval-based analysis derived from DNA methylation or ChIP/ATAC-sequencing. Gene-set analysis tools return, as a result, a list of significant gene-sets. However, while these results are useful for the researcher in the identification of major biological insights, they may be complex to interpret because many gene-sets have largely overlapping gene contents. Additionally, in many cases the result of gene-set analysis consists of a large number of gene-sets making it complicated to identify the major biological insights.

**Results:**

We present GeneSetCluster, a novel approach which allows clustering of identified gene-sets, from one or multiple experiments and/or tools, based on shared genes. GeneSetCluster calculates a distance score based on overlapping gene content, which is then used to cluster them together and as a result, GeneSetCluster identifies groups of gene-sets with similar gene-set definitions (i.e. gene content). These groups of gene-sets can aid the researcher to focus on such groups for biological interpretations.

**Conclusions:**

GeneSetCluster is a novel approach for grouping together post gene-set analysis results based on overlapping gene content. GeneSetCluster is implemented as a package in R. The package and the vignette can be downloaded at https://github.com/TranslationalBioinformaticsUnit

## Background

Modern gene-set analysis (GSA) [1] are standard tools aimed to provide biological insights derived from the list of genes associated with a trait of interest. Tools such as Ingenuity Pathway Analysis (IPA) [2], GREAT [3], GSEA [4], among others, make use of curated collections of gene-sets such as Gene Ontology [5] or KEGG [6] to identify those relevant (statistically significant) gene-sets associated with the trait of interest. However, GSA outcomes may become challenging to interpret when the number of gene-sets identified is very large or if the results from different collections of gene-sets, i.e. different experiments, are combined. An additional challenge appears when identified gene-sets have a high gene content overlap, which could result in nearly identical gene-sets with different functional labels.

Therefore, interpreting the output of gene-set enrichment can be challenging, multiple tools have tried to make the output easier to interpret (Additional file 1: Table 1). The currently available tools utilize gene-sets from specific tools, e.g. David [7] or Go terms, while the output files of custom-curated databases, e.g. IPA and Metacore, are currently not easily compatible with the functionality of the tools. Some tools, like LEGO [8] or GScluster [9] use networking information to elucidate essential information, which requires prior information such as a PPi network, which might not always be available. FGNet [10] establishes links between genes annotated to similar functional terms. Revigo [11] uses semantic based similarities between GO terms. Another major downside of current tools is the focus on a single list of gene-sets, instead of comparing the overlap of gene-sets between several experiments or conditions at the same time. This makes it impossible, or at least cumbersome, to combine results from multiple data sets or tools. Therefore, the current limitations of post GSA analysis are: a lack of unbiased, tools that allow from multiple GSA tools or experiments.

To overcome such limitations, we present **GeneSetCluster**, a tool that consists of three parts. Firstly, **GeneSetCluster** tool harmonizes, making them comparable, outcomes from different gene-set analysis. Secondly, it computes a distance between gene-sets by using the overlap of the content genes. Finally, **GeneSetCluster** uses the distance to cluster the gene-sets with high similarity together into clusters. Those clusters provide the user requires with the reduced set of entities to characterize and these highly similar clusters can be applied to gain insights in the biological information. Because **GeneSetCluster** uses harmonized information of genes directly, this makes **GeneSetCluster** able to use information from any database, across species, and include any custom databases and, we have designed **GeneSetCluster** in a way that enables simple simultaneous analysis of multiple experimental conditions, settings, databases and/or tools.

Briefly, with **GeneSetCluster**, implemented as an R package, we provide an efficient pipeline to process GSA derived gene-sets into clusters of similar gene-sets to facilitate the interpretation of GSA-derived biological insights from one or more experimental conditions and/or tools.

## Implementation

**Fig. 1:**
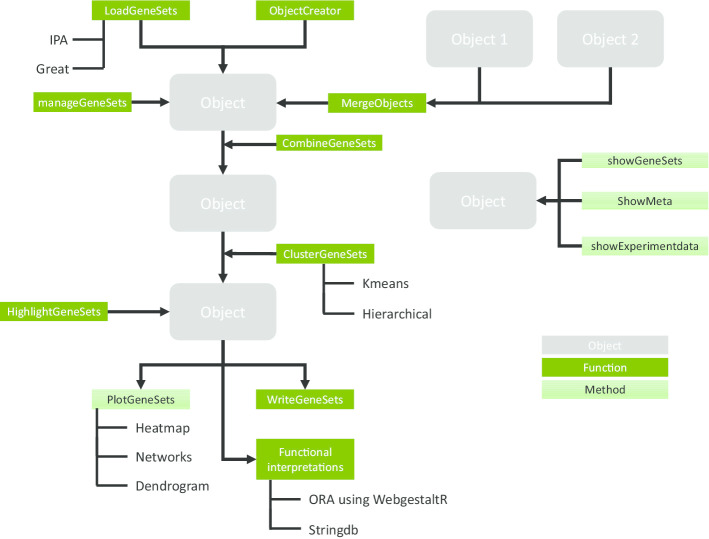
Pipeline of the package. Overview of the standard **GeneSetCluster** pipeline. A *PathwayObject* is created using *LoadGeneSets* or *ObjectCreator*. This is followed by harmonizing and distance calculation (*CombineGeneSets*), deteremine the optimal number of clusters (*OptimalGeneSets*) and clustering (*ClusterGeneSets*). Data is then visualized (*PlotGeneSets*), exported (*WriteGeneSets*) or used for functional interpretations (*ORAperGeneSets/GetSTRINGdbPerGeneSets*)

In **GeneSetCluster**, the gene-set analysis outcomes derived from one or several GSA analysis are combined for a more accurate biological interpretation. **GeneSetCluster** is implemented in R and can be run on any platform with an existing R (version 3.4.4 and above). The package generates a *PathwayObject*, which houses all the information necessary to run the package which gets updated as the analysis progresses. The pipeline starts by loading pathway data into R (Fig. 1) in order to create a *PathwayObject*. For tools such as IPA and GREAT, automatic loading functions have been added (*LoadGeneSets*). Additionally, there is an object creator (*ObjectCreator*), which allows the generation of *PathwayObjects* derived from any GSA analysis or tool, with only minimal information required. This pipeline allows merging several objects, such as loading of data from multiple experiments or data from different tools (*MergeObjects*). If a large number of pathway categories gets loaded, e.g. GREAT output, *manageGeneSets* can help to reduce the number of categories to reduce computational time.

## Processing the gene-sets

### Harmonizing

The first step in the pipeline is to harmonize the data into a common vocabulary and reduce redundancy. This is important for data from different tools, different annotations (gene annotations and/or set annotations) and different experiments. After loading and filtering, the pipeline uses Bitr from the Clusterprofiler package [12] to translate between different biological IDs, e.g. Gene symbols and Ensembl IDs. It uses species information for this conversion, making it possible to compare and/or integrate e.g. mouse and human GSA-derived results.

### Distances

The pipeline then calculates the distance between gene-sets using *CombineGeneSets*. The pipeline default setting is the relative risk (RR), taken from comorbidity statistics [13], using the formula ${RR}_{ij}=\frac{{C}_{ij}/N}{\left({P}_{i}{P}_{j}-{C}_{ij}\right)/N}=\frac{{C}_{ij}N}{{P}_{i}{P}_{j}-{C}_{ij}}$. Where ${C}_{ij}$ is the overlap between molecules of pathway 1 and pathway 2, $N$ is the total number of genes in the experiments, $Pi$ is the molecules of pathway 1 and $Pj$ is the molecules of pathway 2. The other options available are the Jaccard index, which represents percentage overlap, and Cohen’s Kappa, which represents the level of agreement between the gene sets. Moreover, the pipeline allows the user to supply their own distancing function if desired.

### Clustering

To cluster the gene-sets into groups based on the calculated distance, *ClusterGeneSets* allows for two different methodologies: kmeans clustering [14] or hierarchical clustering [15], though custom clustering functions can also be supplied. To determine the optimal number of clusters there is *OptimalGeneSets*, which determines the optimal number of clusters using the elbow, gap or silhouette method. After computing the gene-set clusters, it is possible to highlight clusters for their abundance of genes from a user supplied gene subset, e.g. genes related to reactive oxygen signaling (ROS). This creates a highlighted score. The genes that are in every cluster or unique to the cluster can be explored using GenesPerGeneSet.

### Visualization

**Fig. 2:**
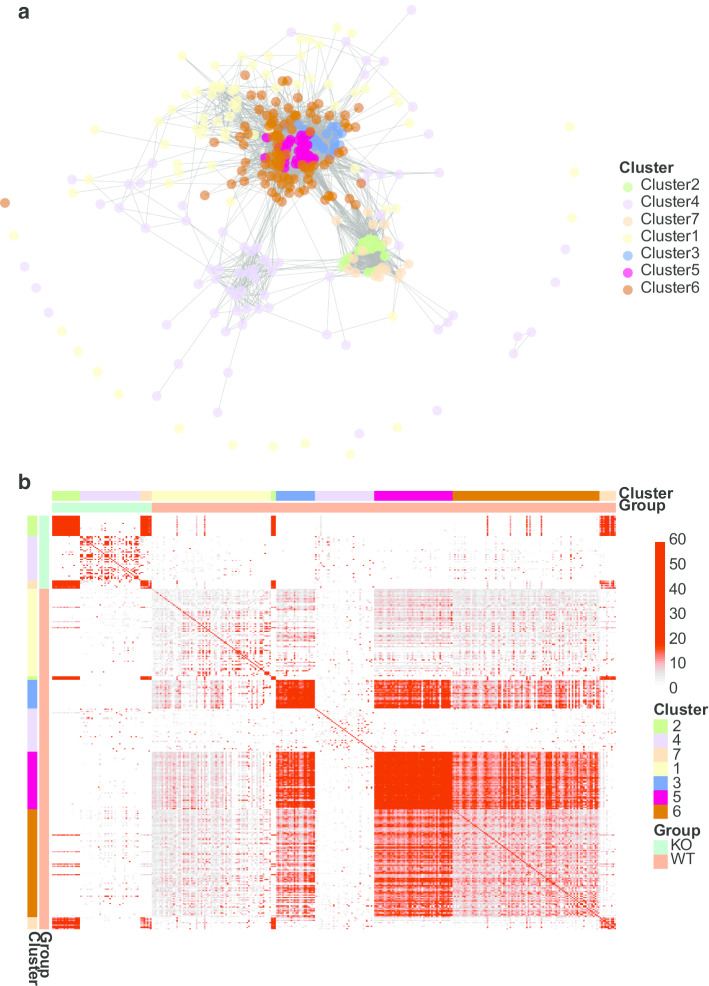
Plotting example data. Output of the *PlotGeneSets* used on IPA canonical pathways clustered with Kmeans into 7 clusters. Data taken from Lund et al. [18]. **a** Networks plot with edges for gene-sets with a distance above 15. **b** Heatmap of the distance scores between them with the RR upper limit set at 70

Following clustering the pipeline can visualize the distance score. Visualization can be either as a network plot using *PlotGeneNetworks* (Fig. 2a), as a dendrogram using *PlotDendrogram* and as a heatmap using *PlotGeneSets* (Fig. 2b). The heatmap uses the pheatmap function and can include the highlighted score as well as overlap of specific molecular signatures in multiple gene-set groups.

### Interpretation

The clusters can be interpreted based on the different labels in the cluster but the package also provides plugins to WebgestaltR (*ORAperGeneSet*) and StringDB (*GetSTRINGdbPerGeneSets* and *plotSTRINGdbPerGeneSets*), which can easily analyze the clusters for either unique or all genes present, which can aid in the biological interpretation.

### Exporting results

After clustering and visualization, the pipeline allows for exporting of all the data, the pathways, the distances calculated and the clusters.

## Practical examples

We have applied this tool in several of our publications. We first used the tool in our EBioMedicine paper in 2019 [16], here we looked for changes in DNA methylation between cases with differing stages of Multiple Sclerosis (MS) and control from 4 different cell types at once, and we clustered our combined into groups of genes. We ended with three major clusters of genes, which we wanted to compare using pathway analysis. After analysing the genes using IPA we found that different clusters displayed gene-sets with similar names, but with different genes enriched, making it difficult to elucidate the different functions between cases and controls. It was only after we compared the different gene-sets using GeneSetCluster, that we could elucidate several clusters of functionally distinct gene-sets between the different disease stages and controls.

We also applied GeneSetCluster in our Nature Communications paper in 2019 [17] where we investigate the effect of dimethylfumurate (DMF) treatment at baseline and 6 months on CD4 and CD14 in the context of MS. Here we found in both cell types different clusters of gene-sets with varying enrichment of Reactive Oxygen Species (ROS) genes, which we hypothesized was related to the effects of the drug. The different clusters with varying levels of ROS has distinct cellular functioning.

## Conclusion

Gene-set analyses are useful tools to summarize major biological trends in a study, however the large number of annotated gene-sets and often a large overlap between them can make it difficult to interpret the results or to compare experiments. We have addressed the need for a method to assess similarity of gene-sets within and between tools and conditions and to cluster them together in an unbiased manner by developing **GeneSetCluster**. **GeneSetCluster** harmonizes different gene-sets and calculates the distance between them to facilitate the functional analysis of gene-set data. **GeneSetCluster** is publically available at https://github.com/TranslationalBioinformaticsUnit/. More information, including a user guide, example script and an extensive wiki, can be found on the github. Furthermore, the github has a link to a step-by-step user guide video on YouTube.

## Supplementary information

**Additional file 1:** **Table S1.** Overview of gene sent enrichment tools.

## Data Availability

Availability of GeneSetCluster R package: https://github.com/TranslationalBioinformaticsUnit/GeneSetCluster. Example GSA data is available in the R package (https://github.com/TranslationalBioinformaticsUnit/GeneSetCluster/tree/master/inst/extdata) with the raw transcriptomic data is available in GEO repository: GSE111385 (https://www.ncbi.nlm.nih.gov/geo/query/acc.cgi?acc=GSE111385).

## Abbreviations

GSA: Gene set analysis
IPA: Ingenuity pathway analysis
RR: Relative risk
MS: Multiple sclerosis
DMF: Dimethyl fumarate
ROS: Reactive oxygen species
